# Dignity and respect during pregnancy and childbirth: a survey of the experience of disabled women

**DOI:** 10.1186/s12884-018-1950-7

**Published:** 2018-08-13

**Authors:** Jenny Hall, Vanora Hundley, Bethan Collins, Jillian Ireland

**Affiliations:** 1 Centre for Excellence in Learning, Bournemouth University, Bournemouth, UK; 2 Centre for Midwifery, Maternal & Perinatal Health, Faculty of Health & Social Sciences, Bournemouth University, Bournemouth, UK; 3 Institute of Clinical Sciences, Faculty of Health and Life Science, University of Liverpool, Liverpool, UK; 4 Occupational Therapy, School of Health Sciences, University of Liverpool, Liverpool, UK; 5 Poole NHS Foundation Trust, Poole, UK

**Keywords:** Disability, Pregnancy, Childbirth, Human rights, Internet survey, Continuity of carer

## Abstract

**Background:**

Despite the increasing number of women with disability globally becoming pregnant, there is currently limited research about their experiences. A national survey of women’s experience of dignity and respect during pregnancy and childbirth raised concerns about the possibility of women with disability having unequal care with overall less choice and control. To address this further we conducted a study to explore the experiences of dignity and respect in childbirth of women with disability.

**Methods:**

The study involved a self-selecting, convenience sample of 37 women who had given birth in the United Kingdom and Ireland and had completed an internet-based survey. Women were identified through online networks and groups of and for disabled parents and for people with specific medical conditions. Data were collected using an online survey tool. Survey data were analysed using descriptive statistics. Thematic analysis was used for open questions.

**Results:**

Despite generally positive responses, just over half of the group of women expressed dissatisfaction with care provision. Only 19% thought that reasonable adjustments or accommodations had been made for them (7/37). When reasonable adjustments were not in place, participants’ independence and dignity were undermined. More than a quarter of women felt they were treated less favourably because of their disability (10/37, 27%). At all points in the pregnancy continuum more than a quarter of women felt their rights were either poorly or very poorly respected; however this was greatest in the postnatal period (11/35, 31%). In addition, more than half of the women (20/36, 56%) felt that maternity care providers did not have appropriate awareness of or attitudes to disability.

**Conclusions:**

Women’s experiences of dignity and respect in childbirth revealed that a significant proportion of women felt their rights were poorly respected and that they were treated less favourably because of their disability. This suggests that there is a need to look more closely at individualised care. It was also evident that more consideration is required to improve attitudes of maternity care providers to disability and services need to adapt to provide reasonable adjustments to accommodate disability, including improving continuity of carer.

## Background

It is estimated that approximately 21% of the population in the United Kingdom (UK) has a disability, with the most common groups of disabilities reported being those associated with mobility, fatigue and mental health [1]. Women are more likely than men to report disability and the prevalence rises with age [1]. Among women of childbearing age the prevalence of disability is believed to be between 6 and 10% [2]. However, identifying the number of women who would be considered ‘disabled’ is challenging, as in most health systems information about women with disability specifically is not gathered [3, 4].

Disability is regularly defined in contexts related to impairments, activity limitations, participation restrictions, and environmental factors [5]. However, the World Health Organization (WHO) definition of disability has recently been revised from a disease focus to one that emphasises health. This change of focus is significant when considering pregnant women with disabilities. In a biopsychosocial model of disability, providers recognise that women with disabilities are knowledgeable about their disability, full partners in decision making and the experts on how their own bodies respond; having had their own lived and individual experience [6]. Several studies highlight the following problems identified by women with disabilities: access; information; communication and choice [2, 7].

There are different ‘models of disability’ or theoretical positions that underpin divergent perspectives on disability such as whether disability is viewed as an individual or social phenomenon. Disability activists, for example, reject the WHO classification of disability and instead propose that while impairment is individual to the person, disability occurs due to barriers within the physical and social environment [8]. It could be argued, therefore, that within a social or rights-based disability model a person is ‘disabled’ by society; that is she is seen as a ‘disabled woman’ rather than a woman who has a disability.

There has been considerable dialogue around access to health and social care for disabled individuals [9], but reports rarely mention disability in relation to care at the time of birth. Studies have examined interventions targeted at improving support for women with disability with children [10] and access to care for women with disability affected by domestic abuse [11], but less is known about the general experiences of maternity care and the issues encountered by women who are disabled. A recent secondary analysis of a national survey conducted in England found that in many areas there was no difference in the care that disabled and non-disabled women received [2]. However, the survey did identify the need for better communication in the context of individualised care. There is some evidence that suggests that women with disability do not feel that staff have adequate knowledge about their needs [12] and health carers have also identified a ‘lack of competence, knowledge and skill’ around disability as well as not recognising that they may not be providing individualised care to women [13].

Patient-centred care, that is compassionate and individualised care, has been the focus of a number of studies and reports. In relation to maternity care, one study used data from a national survey to identify evidence of concerns about care [2]. Redshaw et al.’s study was completed prior to the report from the Mid-Staffordshire public inquiry, which highlighted serious failures in care at one hospital and the lack of a “patient centred culture” across the National Health Service in the UK [14]. Their study also preceded the development of the UK strategy of developing compassionate care in health services, which aimed to put patients first [15]. These studies and reports have had a significant impact on UK practice and are now intended to underpin nursing and midwifery care. The recent National Maternity Services review in England [16] identified that women require care that is individualised to their needs, autonomy in the choices they make and continuity provided by a relationship with a known small group of midwives. Though not focused on women with disability, this highlights that current organisation of services is not meeting all women’s requirements or expectations.

In 2013 Birthrights conducted the first large-scale maternity survey in the UK to focus exclusively on women’s experience of dignity and respect during pregnancy and childbirth. Although the *Dignity in Childbirth Survey* did not set out specifically to examine the experiences of women with disability, the survey findings indicated that the small number of women who identified themselves as disabled appeared to have unequal care with less choice and control over their experience, including less information and reduced choice in pain relief [17]. The survey concluded that further research was needed and with this in mind Birthrights collaborated with Bournemouth University to explore the experience of women with disability throughout pregnancy, childbirth and the first few post-natal weeks (the pregnancy continuum). The study had two consecutive phases: an initial quantitative survey, to identify the experiences of women in the UK and Ireland with physical or sensory impairment during the pregnancy continuum, and a follow-up qualitative study to establish in-depth views and experiences of human rights and dignity in maternity care of a self-selecting group of women. This paper describes the findings from the quantitative survey.

### Aim

The aim of the study was to explore the experiences of dignity and respect in childbirth of women with disability.

## Methods

The study involved a self-selecting, convenience sample of women who had given birth in the UK and Ireland and who completed an internet-based survey. Women were invited to participate through online networks and groups of and for disabled parents, and for people with particular medical conditions (such as arthritis, multiple sclerosis, spinal cord injury and chronic fatigue syndrome). The self-selected sample was therefore drawn from the population of women who identified themselves as having a physical or mobility impairment, sensory impairment (such as impaired vision or hearing) or a long-term health condition that impacts on their daily life (such as chronic fatigue). As the specific needs of women with conditions related to emotional and psychological wellbeing would present different factors, and there are a range of specific perinatal mental health services already in place, this group was not included in the study. Similarly women with learning disabilities were excluded.

The research team recognised that it would be challenging to access a distinct population of women with disability who had experienced the pregnancy continuum. Since sampling from mainstream maternity services would lead to few participants available from a large volume of contact, women were recruited through organisations of and for disabled women / disabled parents and through social media networks. A list was compiled to include umbrella disability organisations, those that focus on one type of disability (e.g. Blind Mums Connect) and those for people with specific medical conditions (e.g. people with spinal cord injury). They were contacted by e-mail and through social media (including Twitter and Facebook) and asked to circulate the link to the online survey. Birthrights and other organisations focusing on maternity care also shared Tweets to inform potential participants of the study.

Data were collected using an open online survey tool delivered through Bristol Online Survey in 2016. The survey was based on the Birthrights’ *Dignity in Childbirth Survey*, which was used with a large population of pregnant women including women with disabilities [17]. The online format of the survey was designed to be accessible for participants who use assistive technology and supplementary information on how to access the survey was provided in a range of formats. There was a total of eight screens (pages) in the survey with an average of four items per page. Respondents were able to review and change their answers through the use of a Back button. The survey link was distributed via social media sites, and through connections via email to groups and charities related to disability. The survey was also available to be answered orally if required, but no one took up this option. The survey contained both open and closed questions relating to dignity, respect, human rights and health equality issues. Questions covered the experience of women during the antenatal, birth and early postnatal periods and related to physical, emotional and human rights experiences. Many free-text boxes were also provided to enable opportunity to respond more fully as required. Neither randomisation of items or adaptive questioning was used within the online questionnaire.

Ethical approval was obtained from Bournemouth University’s Research Ethics Committee. Participant information was available in a range of accessible electronic formats (including large and clear print, screen-reader and assistive technology accessible text; British Sign Language or Irish Sign Language videos would have been provided if required). Consent to participate in the survey was obtained on the landing page where information was provided and individuals were requested to consent by clicking either ‘agree to participate’ or ‘don’t want to participate’. Participation was voluntary, and those who did not consent were directed away from the survey to a page thanking them for their time. Confidentiality was protected by ensuring that the survey did not contain personal identification or information that would identify participants, such as names, email or IP addresses. No incentives were offered for completing the survey.

All data presented in this paper came from the survey. Numerical data were analysed using descriptive statistics. The study was specifically focused on women living in the UK and Ireland; although some international responses to the online survey were received, these were removed prior to data analysis. Thematic analysis was used [18] for open questions, which involved extracting all of the text, open coding and drawing themes. This was completed by the third author and reviewed by the first and second authors. Themes from each question were analysed first, resulting in a small cluster of themes, which were used to provide a summary of the responses to that specific question. Following analysis of all of the open-ended questions, the themes were compared to produce overall themes from the open-ended questions. This enabled a form of constant comparison analysis to be undertaken and differences and discrepancies between responses to questions were explored. While this presentation of the final stage of analysis is not included in this paper, it served to provide nuance and increase trustworthiness of the interpretation of responses to the open-ended questions.

## Results

A total of 46 surveys were completed, however 5 responses were excluded because they came from women based in the United States of America (*n* = 3) and Canada (*n* = 2). A further four participants did not consider themselves to be disabled or Deaf and so these responses were also excluded. This left 37 responses for analysis.

Participants ranged between 21 and 46 years of age with the majority being aged 30–39 years (Table 1). Most women had given birth, but for one woman this was her first pregnancy and she had not yet given birth. Participants were asked how they characterised their primary impairment; most women reported having a physical or mobility impairment. Participants were offered the opportunity to describe their impairment using their own words. The two participants that identified as deaf or hard of hearing simply stated, ‘hearing impaired’ or ‘hard of hearing’. Of the seven blind/visually impaired people, two identified as totally blind with the others identifying that they are partially sighted. The majority of women who identified as having a physical impairment described what would traditionally be classified as musculoskeletal problems, such as arthritis, joint problems and conditions that cause joint hypermobility. Some of these accounts describe how pregnancy exacerbated existing disability due to body changes in pregnancy. On-going health issues were described in less detail, with one participant describing moderate ME/CFS.

**Table 1:** Participant characteristics and care received

		n	%
Age (*n* = 36)	*mean 35.64 (SD 6.1.88)*		
	20–29	7	*19%*
	30–39	19	*51%*
	40–49	10	*27%*
Number of children (*n* = 35)			
	0	1	*3%*
	1	13	*37%*
	2	14	*40%*
	3	5	*14%*
	4	2	*6%*
Primary impairment (*n* = 37)			
	Deaf / hard of hearing	2	*5%*
	Blind / visual impairment	7	*19%*
	Physical or mobility impairment	19	*51%*
	On-going health issue that affects daily life	6	*16%*
	Mental health or emotional issue	3	*8%*
*Antenatal care*			
What kind of antenatal care did you receive? (n = 37)			
	Community midwife only	6	*16%*
	Community midwife and GP	3	*8%*
	Community midwife, GP, and obstetrician	22	*60%*
	Other (specialist team or combination)	6	*16%*
*Birth*			
Place of birth (n = 36)			
	Home	2	*5.5%*
	Stand-alone midwifery-led unit	3	*8%*
	Alongside midwifery-led unit	1	*3%*
	Obstetric unit	28	*78%*
	Theatre	2	*5.5%*
How long ago did you last give birth (in years)? (n = 36)			
	0–2	21	*58%*
	3–5	6	*17%*
	6–10	7	*19%*
	> 10	2	*5%*
Which country were you in when you gave birth? (n = 36)			
	England	20	*55%*
	Scotland	2	*6%*
	United Kingdom	12	*33%*
	Ireland	2	*6%*
*Postnatal care*			
What kind of postnatal care did you receive? (n = 37)			
	Care in hospital	22	*60%*
	Home visit – midwife	27	*73%*
	Home visit – maternity support worker	8	*22%*
	Home visit – Health worker	20	*54%*
	Other (day care, mental health team)	3	*8%*

### The maternity care received

The majority of participants (21/36, 58%) had given birth within the last two years (Table 1). More than two thirds of women (25/37) received shared antenatal care; this was most often shared between the midwife, general practitioner and obstetrician (22/37, 60%). Most women reported that they gave birth in an obstetric unit (28/36, 78%). All women (37) reported receiving some form of postnatal support (participants could choose more than one option) and most indicated that they had support in hospital (22/37) and in the community from a midwife (27/37), and a home visit from a health visitor (20/37).

Participants were generally happy with the support that they received from maternity care providers (Table 2). All women had received care from a midwife in their most recent pregnancy, and 71% were satisfied or very satisfied with that support (25/35). Most women reported satisfaction with general practitioner (20/35, 57%), obstetrician (19/32, 59%) and health visitor (19/34, 56%) support. Fewer reported satisfaction with maternity support worker input (6/20, 33%), but only half of the participants (*n* = 20) answered the question. A number of women stated that they did not know what a maternity support worker was.

**Table 2:** Satisfaction with childbirth experience

	*Very Dissatisfied*	*Dissatisfied*	*Neither*	*Satisfied*	*Very Satisfied*
Satisfaction with support received					
Midwife (n = 35)	*9%*	*14%*	*6%*	*34%*	*37%*
General Practitioner (n = 35)	*6%*	*11%*	*26%*	*31%*	*26%*
Obstetrician (*n* = 32)	*0*	*19%*	*22%*	*28%*	*31%*
Maternity Support Worker (n = 20)	*5%*	*10%*	*55%*	*15%*	*15%*
Health Visitor (*n* = 34)	*6%*	*18%*	*21%*	*32%*	*23%*
Satisfaction with childbirth experience					
Information about services available (n = 36)	*16%*	*14%*	*11%*	*43%*	*16%*
Appropriateness of information for you (n = 37)	*5%*	*40.5%*	*13.5%*	*24%*	*16%*
Extent services were tailored to your needs (*n* = 37)	*13.5%*	*35%*	*19%*	*19%*	*13.5%*
Reasonable adjustments for you needs (n = 37)	*13.5%*	*27%*	*27%*	*13.5%*	*18.9%*
Signposting to other services/local resources(n = 36)	*22%*	*36%*	*17%*	*19%*	*6%*
Extent to which your individuality/preferences were respected (n = 37)	*30%*	*27%*	*11%*	*24%*	*8%*
Overall understanding that service providers showed of your specific situation (n = 37)	*30%*	*27%*	*13%*	*19%*	*11%*
Extent to which your privacy was protected (n = 37)	*3%*	*19%*	*24%*	*35%*	*19%*

Despite generally positive responses, just over half of the women (19/37) expressed dissatisfaction with one or more care providers. The majority of participants (22/37, 59%) were happy with the information about the services available (Table 2); however there was significant dissatisfaction with other aspects of the service. Dissatisfaction was greatest for the statements “*The extent to which your individuality and preferences were respected*” (21/37, 57%) and “*The overall understanding that service providers showed of your specific situation*” (21/37, 57%).

The information from the open-ended questions about the support received comprised of themes about maternity care providers’ awareness and attention to the impact of disability, the need for continuity of carer, the perception of reduced choice or choices being overruled and care providers needing more information. Many of the comments made by participants, particularly those with physical disabilities, suggested that maternity care providers seemed to lack knowledge about disability and how that can influence pregnancy, childbirth and parenting:

*No one understood my disability. No-one knew how to help or who to send me to for support*. (Participant 14 with physical impairment and long-term health condition)

*Service providers had no understanding of specific needs and are only equipped for the mainstream.* (Participant 9 with visual impairment)

*My community midwife was amazing as was my GP. The consultant was unfamiliar with my disability and its implications. The midwife on day of delivery was beyond useless deciding she knew better than specialist of my disability. Anaesthetist was oblivious of my disability and failed to read the notes from my specialist. The labour ward were unaware I was disabled prior to arrival for induction, it took 36 hours for them to get me a toilet frame and told me it was ok because there was 1 grab rail. The registrar decided what was best for me and baby without even considering my disability and its implications. The post labour ward did not provide sufficient space for wheelchair or safe use of crutches. They had a perch stool rather than shower stool which I slipped off the moment it got wet and soapy. Postnatal ward could not meet my physical need so said I should go home. Postnatal were infuriating, they wouldn't take needles out of my hands until I had walked to the toilet, I could not walk without crutches and could not walk on crutches with needles in my hands. Anaesthetist did not listen to what I had to say or to my husband or mother who were there to advocate for me when I was unable.* (Participant 19 with physical impairment)

Two participants specifically highlighted the need for maternity care providers to have knowledge of breastfeeding; both of the participants were blind or partially sighted so it could be that provision of information about breastfeeding for this group is particularly challenging.

Participants, particularly those who experienced pelvic girdle pain or pain due to other disability, commented on how little attention was paid to their experiences of pain and its impact on pregnancy and childbirth, or to how they manage their disability.

*In the hospital I had other midwives. One of them was very dismissive of my PGP. I also found that the obstetrician's team didn't have a clue about PGP. I asked them at the beginning as I had a previous back injury and they said it wouldn't cause a problem. They still didn't acknowledge it even when I was on crutches!* (Participant 3 with physical impairment)

Some participants differentiated between different maternity care providers, finding one provider more helpful than others.

*Midwife and obstetrician couldn't have been better. OT was completely useless.* (Participant 1 with a physical impairment)

*I loved my community midwife but she was the only one who wanted to know how I was feeling about things or if I needed explanations. Everyone else made assumptions, talked about guidelines or looked at monitors.* (Participant 3 with physical impairment)

Women suggested that continuity of carer and follow-through with the same provider was better for them than meeting different maternity care providers throughout their pregnancy continuum. Challenges arose where different maternity care providers were involved.

*The issues were continuity of care. For "my" midwife who knew my history she was great. When she went off work and I saw others, they appeared to neither know nor care.* (Participant 21 with a physical impairment)

*Midwife was fantastic. Due to my disability she decided to make herself fully available to me, I saw only her, didn't have to explain my impairment repeatedly to different people. GP was generally useless, had to be reminded by me what each appointment with him was actually for, kept forgetting to do various tests. HV [Health Visitor] was fine, very 'nosy'!* (Participant 33 with visual impairment)

Two of the responses particularly relate to choice. One participant, who has a physical disability and mental health condition described that ‘I had to fight for the birth I wanted’, whereas another participant, who has a physical disability described her choice to have a caesarean section as being ‘overruled’.

*My Disability is unseen and was not recognised by midwives when in labour. I was put under tremendous pressure to give birth naturally when I had already planned a c section. My baby was breech, I had a dislocated hip and was scared my pelvis would literally snap. This was ignored when I went in to spontaneous labour 3 weeks early. C section was safest option for both of us but midwives know best and were pushing so hard for a natural delivery.* (Participant 23 with a physical impairment)

Another participant described the need to demonstrate her ability to adopt certain positions for her choice of birthplace to be possible, which she describes as ‘insulting’.

*The midwives were fine but I told the obstetrician I didn't want to give birth on the delivery suite and they asked me to physically demonstrate I could get into certain positions that they considered necessary for giving birth. I found that quite insulting. It also undermined my confidence in my body...*

*My midwife antenatally and postnatally was great in community, but the midwives in hospital made me feel like they did not have time for my questions, they told me what hospital guidelines were but I didn't feel like they took into account what I wanted. They spent more time with monitors than actually supporting me. The health visitor dumped loads of leaflets that were supposed to answer my questions but they didn't. If I wanted support or my baby weighed I had to go to clinics but they didn't usually have a health visitor, just a nursery nurse who didn't answer my questions.* (Participant 3 with a physical impairment)

The quote above also highlights the need for continuity of carer and the need for staff to have information to answer women’s questions, an experience shared by other participants. One blind participant described employing an independent midwife to provide care for her second pregnancy due to negative experiences with her first birth.

### Dignity and respect

Participants were split over whether they were treated differently as a result of their disability (Table 3). A third of the women reported that having a disability put them in a high risk category. The comments also give some insight into how being treated differently was perceived. Some women saw different treatment as positive, where they wanted and/or received different treatment to take account of their disability. Other participants said they did not want or expect different treatment as this could lead to them being treated less favourably.

*I feel that my disability was largely ignored. I cope well but continuity of care could have been so much better last time. I had to keep going over the same things to different midwives last time. This time I have just one midwife and my consultant. They know me really well and it's so much better.* (Participant 1 with physical impairment)

**Table 3:** Dignity and respect

	n	%
Do you think that your disability, impairment or health issue led to people treating you differently (n = 37)		
Yes	17	*46%*
No	16	*43%*
Don’t Know	4	*11%*
Do you feel that your disability, impairment or health issue automatically placed you at high risk (n = 37)		
Yes	11	*30%*
No	21	*57%*
Don’t Know / Other	5	*13%*
Do you think that reasonable adjustments or accommodations were made for you (n = 36)		
Yes	7	*19%*
No	24	*67%*
Don’t Know / Other	5	*14%*
Were you told that you were more likely to meet the same health care provider at each of your appointments because of your disability, impairment or health issue (n = 37)		
Yes	4	*11%*
No	30	*81%*
Don’t Know / Other	3	*8%*
Do you feel that communication was good throughout your experience (n = 37)		
Yes	11	*30%*
No	19	*51%*
Don’t Know / Other	7	*19%*
Do you feel that you experienced less favourable treatment because of your disability (n = 37)		
Yes	10	*27%*
No	19	*51%*
Don’t Know	8	*22%*
Do you feel that health care providers had appropriate attitudes to disability (n = 36)		
Yes	9	*25%*
No	20	*56%*
Don’t Know	7	*19%*

*Only one person [treated me differently]: lady giving me epidural though I didn't understand her, and I was answering different questions as a result. In fact I do lip read, but during the procedure I couldn't lip read. She was frustrated and shouted at me. The midwife and my husband had to explain to her that I was hard of hearing. She calmed down... A little bit.* (Participant 6 who is hard of hearing)

*I didn't feel like I was treated any different most of the time which is good.* (Participant 27 with visual impairment)

*I say yes [I was treated differently] in a positive way as everything was done to make my pregnancy and delivery go as smoothly as possible.* (Participant 8 with physical impairment)

*Yes while they do [treat me differently] they often don't know what to offer in support or even operate from charitable model which can be ostracising at times.* (Participant 9 with visual impairment)

*They should have treated me differently - to allow for my situation but didn't appear to.* (Participant 21 with physical impairment)

*At times it is right to be treated different. My disability is unseen and even when I signpost educate and explain, my needs are ignored.* (Participant 15 with physical impairment)

Only 19% of women thought that reasonable adjustments or accommodations had been made for them (7/36). Participants’ disability did not increase their likelihood of being told that they would see the same care provider and just over half the women felt that communication was not good (19/37, 51%). Some of the communication issues related to access to information, such as the way a health professional communicated with a person with a sensory impairment. Adjustments to communication would potentially have resulted in better communication with these participants.

*People did not make the effort to look into my face when speaking which is what I need to fully see what they are saying.* (Participant 2 who is hard of hearing)

*NHS [National Health Service; UK] letters such as scan aps all inaccessible.* (Participant 5 with visual impairment)

Other types of adjustments described by participants included better continuity of carer, so that participants did not need to repeat information about their disability at each visit, additional screenings if required, choice of birth options or a carer being able to stay in the hospital setting. Some participants described needing extra help to care for their baby.

*My community midwife was really on top of everything and even slotted in extra visits when I went past my due date to give me extra sweeps. She was fab. No one else really asked what I wanted or presented options that weren't in guidelines.* (Participant 3 with physical impairment)

*… midwife argued my case for a homebirth due to disability, familiarity etc, some weren't [providing reasonable adjustments] i.e. not being allowed to move in hospital.* (Participant 34 with visual impairment)

*Allowed into birthing pool even though midwife believed it would slow labour down, but I knew if I could get off my knees it would help. So they felt they were humouring me but they listened to me.* (Participant 25 with physical impairment)

*I had my own room on postnatal so my husband could stay but we'd had to travel a long way from home to get the appropriate care so this was a minor consolation.* (Participant 36 with physical impairment)

*after giving birth I found it very difficult to stand and walk due to my disability I would not be able to do it on my own, the care I got in hospital was amazing they let my partner stay with me over night and we were put in a room with a double bed and en-suite so I would have everything I needed near me without any difficulty I could not have been any happier with the care I received* (Participant 30 with physical impairment)

When reasonable adjustments were not in place, participants’ independence and dignity were undermined. For some of the participants with physical and mobility impairments, the reasonable adjustments could have been provision of accessible rooms with assistive equipment to facilitate mobility.

*None [no reasonable adjustments were provided]. I had to remain in bed because my wheelchair couldn't fit in the room. Totally removed my independence.* (Participant 30 with physical impairment)

*Postnatal should have given me a bed with wheelchair access. I should have had immediate access to toilet frame and bath or shower stool.* (Participant 19 with physical impairment)

A quarter of women reported that they felt they were treated less favourably because of their disability (10/37, 27%). In addition, more than half (20/36, 56%) felt that maternity care providers did not have appropriate attitudes to disability (Table 3). These findings from the quantitative analysis are strongly echoed in the qualitative comments, with communication and attitude to or knowledge of disability being the most common and strongest themes. Some women described how the challenges that they faced due to disability were not always recognised or managed appropriately, and as a result the lack of support due to disability resulted in less favourable treatment.

*If I were not disabled none of these things would have been an issue. I feel not meeting those needs means I was treated with less favour due to my disability.* (Participant 19 with physical impairment)

*I was told I couldn't have a water birth in case I couldn't get out of the water in a hurry despite demonstrating at 36 weeks I could do it unaided. This made me really cross as what would they do if someone collapsed in the pool anyway.* (Participant 36 with physical impairment)

**Fig. 1:**
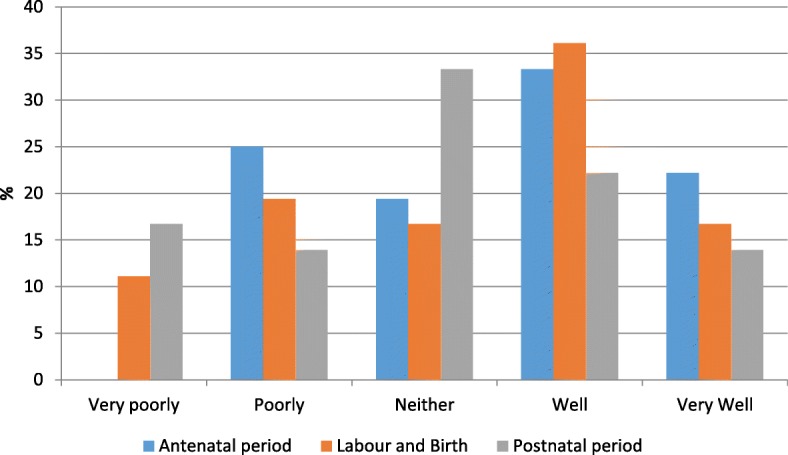
How well were your rights respected?

Participants were asked how well they thought that their rights and their dignity were respected during pregnancy, labour and birth and the postnatal period. More than a quarter of women felt that their rights were either poorly or very poorly respected (Fig. 1: How well were your rights respected). Several participants described their choices over care being limited, that they were not listened to and that their suggested forms of support were not available. When analysing the text, it is noteworthy that the term ‘allowed’ is frequently used, suggesting a power differential where the service providers are ultimately making decisions, allowing or disallowing women’s choices.

*not allowed birthing ball, not allowed to walk around etc* (Participant 34 with visual impairment)

*I wasn't allowed to go to low risk centre despite my disability not affecting my capacity to give birth. Problems in pregnancy weren't addressed i saw a specialist but too late then needed my care transferring urgently but this took over a week introducing another significant delay and has left me with long term problems (daughter is fine). Because my problems were related to my disability I felt they weren't addressed with the same sense of urgency as with pregnancy related problems.* (Participant 36 with physical impairment)

*They would not allow my carer to stay overnight* (Participant 32 with physical impairment)

**Fig. 2:**
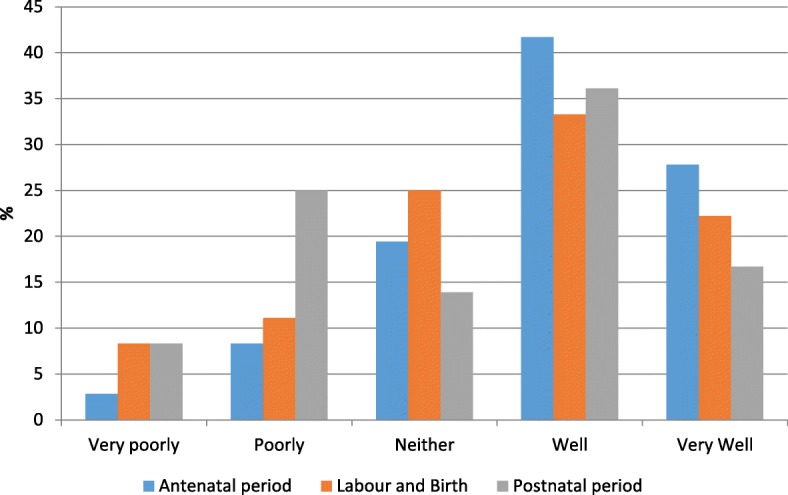
How well was your dignity respected?

Slightly fewer women felt that their dignity was either poorly or very poorly respected in the antenatal period (11%) or during labour and birth (19%); however a third felt that their dignity was either poorly or very poorly respected in the postnatal period (33%) (Fig. 2: How well was your dignity respected?). Dignity seemed to be interpreted as being able to make choices by some women. Other women described undignified care as when their individuality (and disability) was not respected and they felt to be considered to be an ‘annoyance’ by service providers. The key themes arising from the perception of dignified care echo comments from the earlier parts of the survey: women want to be listened to, taken seriously and to have their wishes respected.

*I felt that staff were annoyed by my requests, and that they behaved as if my physical limitations were an inconvenience.* (Participant 32 with physical impairment)

*I was treated as though I was being dramatic. The communication was poor. All of my options if there were any were not explained.* (Participant 17 with physical impairment)

*I find being in a wheelchair means I am regularly not listened to. My husband or mum are asked questions instead of me. When the professional doesn't like what I have to say they looked to my mum or husband to put me in my place (at least that is how it felt).* (Participant 19 with physical impairment)

*I was told I was a health and safety risk, people didn't speak directly to me, felt smothered.* (Participant 34 with visual impairment)

### Improving the experience

Participants were asked to provide advice and suggestions for maternity care providers to improve the experience of women with disability during pregnancy, childbirth and early parenting. Women overwhelmingly highlighted the importance of communication, particularly listening, and respecting a woman’s wishes, her difference and that she knows her body and disability best. Continuity of care was raised by many of the women. In addition, participants highlighted the importance of learning about disability and having a better understanding of a condition, particularly if it is likely to be exacerbated in pregnancy, and to read women’s notes. The below quotes illustrate this:

*Listen to what women tell you about what they want and ask them if they can do things, don't request them to. Don't tell them what the policies are without explaining how you can adapt them or why they are recommended in that way.* (Participant 3 with physical impairment)

*Listen to the individual. I know my needs and limitations better than anyone else* (Participant 34 with visual impairment)

*Ask on first visit what supports are required an put a plan in place to meet needs that is on file and reviewed and updated regularly which will be available to all health care professionals at the front of file. This will ensure that people with a disability are not constantly explaining their needs. Staff also need to be trained in equality and a rights based model to disability. (*Participant 9 with visual impairment)

*Remember every mum is different whether disabled or not.* (Participant 35 with visual impairment)

*Each woman is different as is each baby. If a woman says she's in pain she invariably is. Just because the general advice following a section is to be as mobile as possible that doesn't mean it is possible for everyone and just because some women feel very little pain post section doesn't mean that those who do suffer are weak or less deserving of your support. Please tailor your care accordingly and listen to what you are being told* (Participant 16 with physical impairment)

*Research medical conditions before you try to treat. If you are told something about the individuals needs/condition, make a note of it and ensure all are aware. Do not say you know what someone feels or needs unless you have been in the exact same position as them. (*Participant 19 with physical impairment)

*If a patient has a syndrome please have a quick google or look at the charity website associated with the condition. In 2 minutes you'll be able to see the main issues associated with the condition, which aren't always what you would expect. Patients know you are unlikely to be an expert in their condition, but they do expect you to know what it is.* (Participant 24 with physical impairment)

*They need to have more detailed understanding of the variety of disabilities or even have some equality champions who can be called upon to liaise with mum (*Participant 30 with physical impairment)

*Put yourself in my shoes and figure out how to help rather than follow the standard path. Make an effort to understand how my disability affects me - I'm not asking for extra assistance to be awkward but to try and create a circumstance I can cope with.* (Participant 37 with long-term medical condition)

*Think 'can do' rather than can't!* (Participant 8 with physical impairment)

Some participants also noted that the staffing levels meant that there was not enough time to meet their needs and that for women with disability, additional support and appointments may be needed*.*

*I think if the mother is experiencing any kind of difficulty they should automatically be offered extra midwife appointments and more emotional support.* (Participant 13 with physical impairment)

*To allow women to labour in their own time and accept that refusing drugs is not about being stoic but more about accepting sensitivities to chemicals.* (Participant 25 with physical impairment)

## Discussion

This study used an on-line survey to seek the experiences of pregnancy, childbirth and early parenting of women with disability. While there was no comparison with non-disabled women in the current study, some of the women did describe less positive experiences, which replicates findings of other studies [2, 7, 19], including that undertaken by Birthrights [17]. However, it is important to acknowledge that this was a self-selecting sample and as a consequence it is open to selection bias. That is, it could be argued that women who responded to the invitation to participate might be more likely to have previously experienced poor maternity services and therefore were more motivated to provide feedback. Selection bias is a known problem with online surveys, particularly where a link is circulated to interested groups [20]. The small sample size, and the fact that it contained a high proportion of women with certain types of physical disability, means that it is unlikely to be representative of the population of women with disability as a whole. In the future, more specific sampling of a smaller population – for example, women with specific impairment types – may yield more representative results.

In spite of these limitations, the women’s accounts clearly point to aspects of care that could be improved. Indeed, we believe that this is the first study that has specifically looked at the experiences of dignity and respect in relation to childbirth of women with disability. Lowe has noted that ‘dignity in health care is defined as encompassing respect and autonomy’ [21] p137). Our findings indicate that this was sometimes missing in the interactions between women and their maternity care providers. Women explicitly indicated feeling that they were not being listened to and that they had fewer choices and this affected their sense of dignity. In a review of complaints in relation to UK maternity care, Morad et al. found that poor communication, and specifically a failure to listen or consider the woman’s viewpoint, was the primary cause for complaint [22].

Morad et al. [22] highlight that dignity can be maintained when women are treated as individuals and when they can build a relationship of trust with their maternity care providers. For the women with disability in our study this point was key. Women reported that a lack of continuity of carer caused them significant problems as their condition was not understood and adjustments were not made. Women reported needing to repeat themselves again and again and their wishes, as discussed and agreed with one maternity care provider were not followed through by another. The most often repeated theme from the open-ended questions was that women felt that they were not being listened to and that this had the potential to reduce their choices and made them feel like they had less control. These findings echo previous research about the experience of women with disability [12]. Others have suggested that women with disability may experience greater continuity of carer or more ante-natal care [2], but there was limited evidence of this in our study.

More concerning was the fact that more than a quarter of women felt that their rights were poorly or very poorly respected; a quarter felt they were treated less favourably because of their disability and more than half (56%) felt that maternity care providers did not have appropriate attitudes to disability. Discriminatory behaviour and lack of respect was also highlighted in the national survey completed for the Care Quality commission [19]. A few participants explicitly described situations in which they felt their dignity was undermined, for example being asked to demonstrate being able to get into a specific position and being asked to mobilise when they felt unable to do so due to physical disability. Participants criticised the lack of knowledge that maternity care providers had about disability and its impact on pregnancy, childbirth and parenting, highlighting that this was, for some, offensive and made them feel less confident in themselves. The call for all women to receive respectful maternity care is not new, but it has received added impetus with the publication of *the Respectful Maternity Care Charter* [23] and Birthrights’ *Dignity in Childbirth Survey* [17]. Our findings highlight the urgent need for maternity care providers to develop better understanding and approaches when supporting women with disability. Additional education for maternity care providers should include information about different approaches to disability and highlight the need to listen to the woman to understand her unique disability experience.

However, there is evidence that simply implementing the recommendations of a recent maternity services review [16] would address many of the challenges in England. *Better Births* highlights the importance of personalised care, which is woman-centred, with opportunity for choice and control, and continuity of carer. Adapting services to provide continuity of carer for all women would make it more likely that women with disability have the appropriate accommodations and support in place.

Continuity of carer is particularly important as only 19% of the women in our study described having the reasonable adjustments that they are legally entitled to receive. Women described inadequate physical environments, space and equipment to cater for physical disability in ante-natal, labour and post-natal facilities, thus reducing their access to services or the dignity with which these could be used. While environments may be universally challenging in terms of protecting the dignity of women, the experiences of women in the current study suggest that, particularly for a woman with a physical disability, inadequate environments can pose additional challenges.

## Conclusion

This is the first study that has specifically looked at the experiences of dignity and respect in childbirth of women with disability. More than a quarter of the women in the study felt that their rights were poorly respected and that they were treated less favourably because of their disability. It was also evident that more consideration needs to be made to improve attitudes of maternity care providers to disability, and services need to adapt to provide reasonable adjustments to accommodate disability, including improving continuity of carer.

## Abbreviations

UK: United Kingdom
WHO: World Health Organization
